# Disruption of mutated BRAF signaling modulates thyroid cancer phenotype

**DOI:** 10.1186/1756-0500-7-187

**Published:** 2014-03-28

**Authors:** Elyse K Hanly, Shilpi Rajoria, Zbigniew Darzynkiewicz, Hong Zhao, Robert Suriano, Neha Tuli, Andrea L George, Robert Bednarczyk, Edward J Shin, Jan Geliebter, Raj K Tiwari

**Affiliations:** 1 Department of Microbiology and Immunology, New York Medical College, Valhalla, New York 10595, USA; 2 Department of Pathology, New York Medical College, Valhalla, New York 10595, USA; 3 Department of Otolaryngology, New York Eye and Ear Infirmary, New York, New York 10003, USA

**Keywords:** Thyroid cancer, BRAFV600E mutation, PLX4032, MAPK signal transduction pathway, Targeted therapy, Kinase inhibitors

## Abstract

**Background:**

Thyroid cancer is the most common endocrine-related cancer in the United States and its incidence is rising rapidly. Since among various genetic lesions identified in thyroid cancer, the BRAFV600E mutation is found in 50% of papillary thyroid cancers and 25% of anaplastic thyroid cancers, this mutation provides an opportunity for targeted drug therapy. Our laboratory evaluated cellular phenotypic effects in response to treatment with PLX4032, a BRAFV600E-specific inhibitor, in normal BRAF-wild-type thyroid cells and in BRAFV600E-positive papillary thyroid cancer cells.

**Methods:**

Normal BRAF-wild-type thyroid cells and BRAFV600E-mutated papillary thyroid cancer cells were subjected to proliferation assays and analyzed for cell death by immunofluorescence. Cell cycle status was determined using an EdU uptake assay followed by laser scanning cytometry. In addition, expression of proteins within the MAPK signal transduction pathway was analyzed by Western blot.

**Results:**

PLX4032 has potent anti-proliferative effects selectively in BRAF-mutated thyroid cancer cells. These effects appear to be mediated by the drug’s activity of inhibiting phosphorylation of signaling molecules *downstream* of BRAF within the pro-survival MAPK pathway. Interestingly, PLX4032 promotes the phosphorylation of these signaling molecules in BRAF-wild-type thyroid cells.

**Conclusions:**

These findings support further evaluation of combinational therapy that includes BRAFV600E inhibitors in thyroid cancer patients harboring the BRAFV600E mutation.

## Background

Thyroid cancer is the most common endocrine-related cancer in the United States. Its incidence has been rising for the past few decades and is predicted to double by 2019 [1-3]. Based on morphological variation, thyroid cancer can be classified as papillary, follicular, medullary or anaplastic. Papillary thyroid cancer (PTC) accounts for over 80% of all thyroid carcinoma. Most of the thyroid cancers are believed to originate in single cells due to mutations that confer growth advantages including increased proliferation, impaired apoptosis, enhanced angiogenesis, invasion and metastasis. Specifically, mutations causing over-activation of the mitogen-activated protein kinase (MAPK) signaling pathway are found in more than 70% of papillary thyroid cancers and include *BRAF* oncogene activation, *RAS* mutations and *RET* proto-oncogene rearrangement [4]. Among these mutations, a single point mutation involving substitution of glutamate for valine at nucleotide 600 and resulting in mutant BRAF protein (BRAFV600E) is most common. *BRAF* gene encodes a MEK activator within the MAPK pathway, and it is through aberrant protein signaling that genetic mutation in the *BRAF* gene leads to PTC in more than one-third of all cases [5-8].

Treatment of thyroid cancer, although dependent on the stage and type of cancer, usually involves complete or partial thyroidectomy, radioactive iodine (I^131^) therapy, and hormone replacement therapy. Despite available treatment options, twenty to thirty percent of patients develop recurrent thyroid cancer at least once in their lifetime. In addition, a small subset of patients develop advanced, metastatic disease and face limited treatment options after traditional therapy fails, demonstrating the need for therapeutic advances. As a potential therapeutic target, the BRAF mutation plays a central role in promoting aggressive phenotype of thyroid cancer and is associated with worse prognosis. Mutation in BRAF correlates with advanced stage, lymph node metastasis, extrathyroidal extension, as well as resistance to traditional radioiodine therapy in papillary thyroid cancer [9,10]. Guerra et al. found that greater percentages of BRAFV600E alleles within papillary thyroid tumors resulted in poorer disease outcome [11]. In addition, BRAFV600E mutation has recently been correlated with significantly increased cancer-related mortality, as mortality was 5.3% in patients positive for the BRAF mutation and only 1.1% in those without the mutation [12].

As a serine-threonine protein kinase, BRAF plays an important role within the MAPK signaling pathway. Normal activation of this well-studied pathway involves growth factors binding to a variety of cellular receptors including Receptor Tyrosine Kinases (RTKs) followed by activation of small G protein, RAS [13]. RAS recruits proteins to the membrane to cause activation of RAF, which in turn phosphorylates MEK. In cascade –like fashion, MEK phosphorylates ERK (MAPK), a kinase with over 160 downstream targets regulating diverse cellular processes such as growth, cell cycle, differentiation, survival, and apoptosis [13]. Out of three RAF isoforms (ARAF, BRAF, and CRAF), BRAF is the most potent contributor to the MAPK pathway and is the only isoform commonly mutated in human cancer [14]. BRAFV600E mutation disrupts hydrophobic interactions within the BRAF protein, allowing for a catalytically active conformation which continuously phosphorylates MEK, independent of upstream signals [15]. This specific genetic mutation leading to excessive activation of the MAPK pathway accounts for 90% of all cancer-related BRAF mutations and is found in about half of all papillary thyroid cancers and one fourth of anaplastic thyroid malignancies [12,15]. High prevalence of genetic mutations causing altered signaling pathways in human cancer has spurred development of targeted drug therapy focusing on inhibition of intracellular kinases such as mutated BRAF, which is also commonly found in melanoma, ovarian, and colorectal cancers [15].

This targeted drug therapy program identified PLX4032 (Vemurafenib), a small molecule inhibitor which selectively binds to the ATP binding pocket of mutated BRAFV600E, inhibiting its ability to signal within the MAPK pathway. One of the drug’s most appealing characteristics is its selectivity towards BRAFV600E-positive tumors. Theoretically, mutated cancer cells are highly dependent on BRAF signaling and are unable to survive treatment while cells expressing only wild-type BRAF remain unaffected. Potent anti-tumor activity of PLX4032 was demonstrated in BRAFV600E-positive malignant melanoma patients, as the majority of these patients experienced partial or complete tumor regression [16]. In melanoma studies, PLX4032 treatment reduced relative risk of death by 63% and relative risk of death or disease progression by 74% compared to standard treatment with dacarbazine [17]. Clinical studies involving PLX4032 and thyroid cancer are currently underway. In a Phase I trial, PLX4032 treatment led to partial reduction of tumor size in one patient out of three with metastatic papillary thyroid cancer [18]. Interestingly, it was discovered that PLX4032 acts to promote MAPK signaling in BRAF-wild-type cells by binding to one protomer in a RAF dimer and transactivating the other protomer after upstream RAS signaling [19-21]. This phenomenon may account for side effects of the drug, including promotion of squamous cell carcinoma.

In this study, we explore how inhibition of BRAFV600E leads to phenotypic changes in thyroid cells and demonstrate the selectivity of PLX4032-mediated inhibition in thyroid cells. Although the study is limited to one papillary thyroid cancer cell line and is not representative of all thyroid cancer cell lines harboring the BRAFV600E mutation, we present findings which characterize the interaction between a commonly mutated signal transduction molecule and a specific inhibitor.

## Methods

### Cell culture

Two thyroid cell lines in this study include a human normal transformed line (Nthy-ori 3–1) and a human papillary thyroid cancer cell line (BCPAP). Dr. Norman L. Eberhardt (Mayo Clinic, Rochester, MN) generously gifted the Nthy-ori 3–1 cell line and BCPAP was purchased from DSMZ in Braunschh, Germany. Both Nthy-ori 3–1 and BCPAP were cultured in Roswell Park Memorial Institute (RPMI)-1640 (Mediatech, Hemdon, VA) supplemented with 10% fetal bovine serum (FBS) (Atlanta Biologicals, Lawrenceville, GA), penicillin 10,000 IU/mL, streptomycin 10,000 μg/mL (Mediatech), and 2 mM L-glutamine (Mediatech).

### XTT cell proliferation assay

Five thousand cells in 200 μL medium per well were plated onto a 96-well plate for each cell line and allowed to adhere overnight. Complete media in each well was replaced with 200 μLmedia containing 5% charcoal dextran-treated FBS and PLX4032 at concentrations of 0 μM, .5 μM, 1 μM, 5 μM, 10 μM, 20 μM, and 50 μM. Plates were incubated at 37°C for 48 hours. After incubation, XTT was added to each well at 1 mg per mL (plus 5 μL/mL phenazinemethosulfate) and 50 μL per well. Plates were read at 450 nm and a reference of 630 nm. Mean OD values for each concentration of PLX4032 were plotted and used to calculate percent survival compared to the untreated control. IC_50_ value is the concentration corresponding to fifty percent survival.

### Trypan blue exclusion assay

Nthy-ori 3–1 and BCPAP cells were harvested using 0.25% trypsin and plated in six-well culture plates at 500,000 cells in 2 mL media per well. Cells were allowed to adhere overnight and then starved for 24 hours by replacing complete media with phenol-red-free RPMI containing 5% charcoal dextran-treated FBS. Cells were left untreated or treated with 10 μM PLX4032 for 12, 24, and 36 hours. At the end of each time exposure, cells were harvested using 0.25% trypsin and stained with 0.4% trypan blue solution (Sigma). Numbers of cells unable to take up stain were counted as viable cells.

### Immunofluorescence staining

Nthy-ori 3–1 and BCPAP cells were harvested and seeded onto eight-well chamber slides at a density of 10,000 cells in 250 μl per well. Cells were allowed to adhere overnight and then starved for 24 hours by replacing complete media with phenol-red-free RPMI containing 5% charcoal dextran-treated FBS. Cells were left untreated or treated with 10 μM PLX4032 for 36 hours. Adherent cells were stained with FITC-Annexin V, Ethidium Homodimer III and Hoechst 33342 according to experimental procedures of the Apoptotic/Necrotic/Healthy Cells Detection Kit (PromoKine, Germany). Images were captured using Axiovision Rel 4.8 on the Axiovert 200 M microscope (Carl Zeiss MicroImaging Inc., Thornwood, NY).

### EdU flow cytometry assay

Cells were harvested and seeded onto eight-well chamber slides at a density of 10,000 cells in 250 μl per well. As described earlier, cells were allowed to adhere to slides overnight and then starved for 24 hours in phenol-red-free RPMI containing 5% charcoal dextran-treated FBS. Cells were left untreated or treated with 10 μM PLX4032 for 36 hours. Cells were incubated with 20 μM Click-It™ EdU (Click-iT™ EdU Flow Cytometry Assay Kit from Invitrogen) for one hour at 37°C. Cells were then fixed using 4% paraformaldehyde for ten minutes at room temperature and then permeabilized using 0.2% Triton-X for ten minutes at room temperature. The Click-It™ reaction cocktail was used to detect EdU according to Invitrogen protocol, using Alexa Fluor 488 dye. Cells were also stained with DAPI for 15 minutes at room temperature. Intensity of maximal pixel and integrated fluorescence of detected EdU was measured by Laser Scanning Cytometry (LCS) (iCys; CompuCyte, Westwood, MA) [22].

### Western blot analysis

Nthy-ori 3–1 and BCPAP cells were allowed to grow until 70% confluence. Complete media was replaced with 5% charcoal dextran-treated FBS-containing media for 24 hours and cells were treated with 5 μM and 10 μM PLX4032 for 24 hours. Cell samples were subjected to radioimmunoprecipitation assay buffer (50mM Tris–HCl [pH 7.4], 150mM NaCl, 0.2% sodium deoxycholate, 0.1% sodium dodecyl sulfate [SDS], 0.5% NP40, and μM Pefabloc) and vortexed every 5 minutes for 30 minutes over ice. Whole cell lysates were centrifuged at 14,000 rpm for 30 minutes at 4°C. Supernatants were collected and volumes containing 10 μg protein per sample were used for 12% SDS-polyacrylamide gel electrophoresis as in our prior studies [23,24]. Proteins on gels were transferred to Immobilon-P membranes using 220 mA for 2 hours in a transfer chamber. Membranes were blocked using 5% powdered milk in TBST (10 mM Tris–HCl, pH 7.5, 200 mM NaCl, 0.05% Tween-20) for 2 hours on a shaker at room temperature. Membranes were incubated overnight in primary antibodies including MEK1/2, phospho-MEK1/2, p44/42 MAPK (Erk1/2), phopho-p44/42 MAPK (Erk1/2), mTOR, and phopho-mTOR (Cell Signaling Technology). After 3 TBST washes of 5 minutes each, membranes were incubated with secondary antibody for 2 hours at room temperature. Membranes were washed again in TBST (four 10 minute washes) and then developed by enhanced chemiluminescence (Thermo Scientific) and detected on X-ray film.

### Statistical calculation

Statistical significance was determined using the paired Student’s *t* test and a *p* value ≤ 0.05 to reject the null hypothesis.

## Results

### BRAF-mutated papillary thyroid cancer cells are more susceptible to growth inhibition by PLX4032 in comparison to normal wild-type thyroid cells

**Figure 1:**
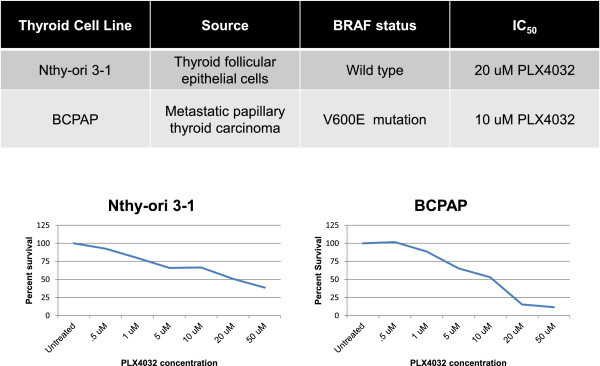
**Characterization of thyroid cell lines.** Nthy-ori3-1 cells are normal, immortalized thyroid cells expressing wild-type BRAF. BCPAP cells are papillary thyroid cancer cells and are heterozygous for the BRAFV600E mutation. Results of an XTT cell proliferation assay demonstrate percent of surviving cells after treatment with various concentrations of PLX4032. An IC_50_ of 10 μM PLX4032 was established for BCPAP cells and an IC_50_ of 20 μM PLX4032 was established for Nthy-ori3-1 cells.

Two thyroid cell lines were chosen to study selective response to RAF inhibitor, PLX4032, based on BRAF mutation status. Nthy-ori 3–1 are normal transformed thyroid cells with wild-type BRAF and BCPAP are papillary thyroid cancer cells harboring the BRAFV600E mutation. Using a XTT cell survival assay with varying concentrations of PLX4032, we observed that cell survival decreased with increasing doses of PLX4032 and almost total loss of cell viability occurred at 20 μM PLX4032 in BCPAP cells. In contrast, at the highest concentration of PLX4032 tested (50 μM), over 25% of Nthy-ori 3–1 cells remained viable (Figure 1). An IC_50_ dose of 20 μM was established for Nthy-ori 3–1 and 10 μM for BCPAP, indicating that our normal thyroid cells are twice as resistant to growth-inhibiting effects of PLX4032 compared to the more susceptible BRAF-mutated cancer cells. Based on the IC_50_ value of PLX4032 for BCPAP cells, 5 μm and 10 μM PLX4032 were used as investigatory concentrations of PLX4032 for our experiments.

### PLX4032 causes apoptosis selectively in BRAF- mutated papillary thyroid cancer cells

**Figure 2:**
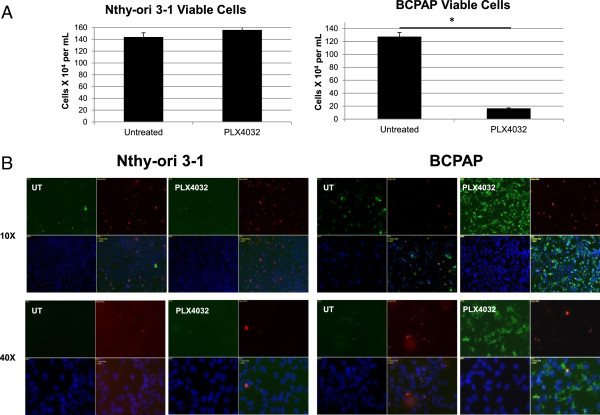
**PLX4032 causes apoptosis selectively in mutated papillary thyroid cancer cells. A)** Nthy-ori3-1 and BCPAP cells were plated in six-well culture dishes in the presence of 10 μM PLX4032 for 36 hours. Viable cells were counted using a trypan blue dye exclusion assay. Asterisks indicate statistically significant differences (p < .05) between control and treated samples. **B)** Nthy-ori 3–1 and BCPAP cells were treated with 10 μM PLX4032 for 36 hours and apoptotic and necrotic cells were visualized by immunofluorescence. Red staining is indicative of necrotic cell death as a result of cellular insult and membrane disruption and green staining marks apoptotic cells that express Annexin V on their outer membranes. (Blue: Hoechst 33342, Red: Ethidium Homodimer III, green: FITC-Annexin V).

Multiple cell-based assays were performed to evaluate the effects of PLX4032 on functional markers of MAPK inhibition including proliferation, survival, cell cycle and apoptosis**.** To evaluate effects on proliferation and survival ability, viable cells were counted after a 36 hour treatment period using a trypan blue exclusion assay. Within BCPAP cells, PLX4032-treated wells contained significantly fewer live cells compared to untreated groups, reaching only about 15% of the number of cells found in untreated groups (Figure 2a). The Nthy-ori 3–1 cells appeared resistant to PLX4032 in terms of cell proliferation, as the numbers of live cells counted in the treatment group were not changed significantly from numbers counted in the untreated wells (Figure 2a). As PLX4032 treatment caused cell death selectively in BRAFV600E-positive BCPAP cells, a further experiment to distinguish between apoptotic and necrotic cell death was conducted. Immunofluorescence revealed that PLX4032 causes apoptotic cell death rather than necrotic cell death in BCPAP papillary thyroid cancer cells and does not appear to contribute to cell death in non-mutated Nthy-ori 3–1 thyroid cells (Figure 2b).

### PLX4032 inhibits nucleotide incorporation selectively in BRAF-mutated papillary thyroid cells

**Figure 3:**
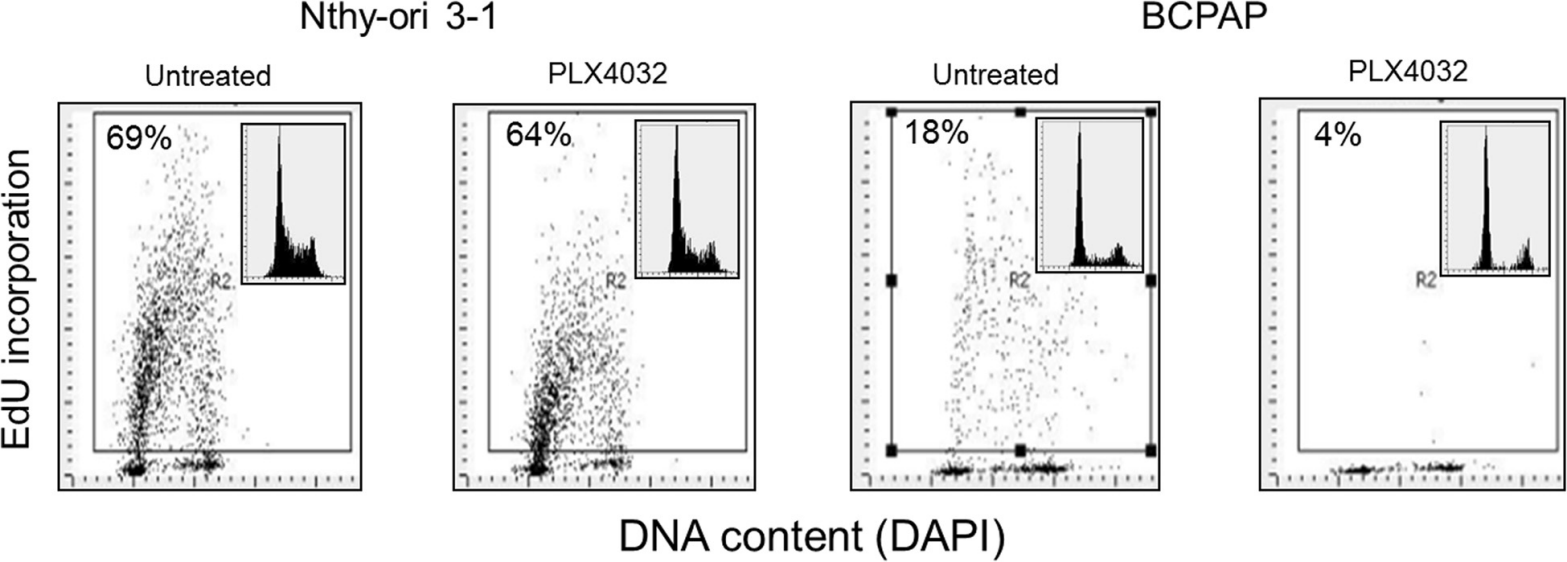
**PLX4032 inhibits incorporation of EdU selectively in mutated papillary thyroid cells.** Nthy-ori 3–1 and BCPAP cells were treated with PLX4032 for 36 hours and then exposed to EdU for 1 hour. The incorporated EdU was detected using the “click chemistry” approach [25], cellular DNA was counterstained with DAPI and intensity of cellular fluorescence was measured by laser scanning cytometry. The data are shown as bivariate (DNA content versus EdU incorporation) scatterplot distributions; insets show DNA content frequency histograms from the respective cultures. Cells positive for EdU were gated and their percentage is shown in the respective panels.

Since PLX4032 has been shown to induce arrest in G1 phase of the cell cycle in BRAF-mutated melanoma cells, we determined whether this effect also occurred in our thyroid cell lines. Analysis of cell cycle status in BRAF-wild-type thyroid cells as well as BRAF-mutated papillary thyroid cancer cells after treatment with PLX4032 was performed by directly measuring DNA replication and assessing cell cycle distribution. In this assay, incorporation of EdU, the DNA precursor, was measured by laser scanning cytometry as an indicator of cells actively synthesizing DNA and in S-phase of the cell cycle. PLX4032 treatment diminished EdU uptake in the BCPAP cells and essentially had no effect on EdU incorporation in normal thyroid cells (Figure 3). Thus, PLX4032 halts DNA synthesis selectively in the BRAF-mutated papillary thyroid cancer cells. Furthermore, as evident from the DNA content frequency histograms, the number of proliferating cells in S and G2/M phases of the cell cycle was much lower in the case of BCPAP cells compared to Nthy-ori 3–1 cells. Collectively, these data suggest that PLX4032 exhibits potent anti-proliferative effects in BRAFV600E-positive thyroid cancer cells.

### PLX4032 selectively downregulates phosphorylation of signaling targets in BRAFV600E-positive thyroid cells

**Figure 4:**
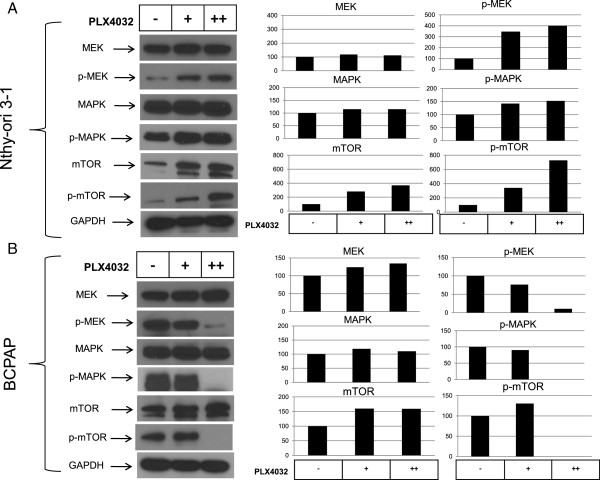
**PLX4032 selectively inhibits phosphorylation of signaling targets in BRAFV600E-positive thyroid cells. A)** Whole cell protein was collected from Nthy-ori 3–1 cells that were treated for 24 hours with 5 μM (+) or 10 μM (++) PLX4032. Protein samples (10 μg per lane) were subjected to SDS-PAGE followed by western blot analysis for MEK (46 kD), p-MEK (45 kD), MAPK (42/44 kD), p-MAPK (42/44 kD), mTOR (289 kD) and p-mTOR (289 kD). GAPDH (37 kD) was used as a loading control. Quantification of Western blots by densitometry was performed using ImageJ and bar graphs represent percent expression compared to the untreated. **B)** BCPAP cells were treated and collected, whole cell lysates resolved by SDS-PAGE and analyzed by western blot in the same way as Nthy-ori 3–1 cells in **A**.

Several groups have demonstrated that mutation-specific BRAF inhibitors such as PLX4032 will selectively inhibit MAPK signaling in BRAFV600E-mutated cells and will activate MAPK signaling in wild-type BRAF cells. To investigate the effects of PLX4032 on signaling within the MAPK pathway in our thyroid cells, we treated non-mutated Nthy-ori 3–1 cells and BRAFV600E-positive BCPAP cells with 5 μM and 10 μM PLX4032 and analyzed protein expression by western blot. The phosphorylation of MEK, MAPK, and mTOR proteins was inhibited by 10 μM PLX4032 treatment in the mutated BCPAP cells (Figure 4b). In contrast to the effect seen in BCPAP cells, PLX4032 treatment of both 5 μM and 10 μM upregulated expression of these phosphorylated proteins in BRAF-wild-type Nthy-ori 3–1 thyroid cells (Figure 4a).

## Discussion

In the past decade or so, biological discoveries including the high prevalence and critical role of genetic mutations in cancer cells have led to the new concept of targeted molecular therapy. Among potential therapy targets, mutated or overexpressed kinases were recognized early on due to their ability to enhance tumor-promoting phenotypes through signal transduction pathways [26]. Small molecule kinase inhibitors can be designed to be specific for cancer cells, and, in general, non-cancerous cells are better able to survive kinase inhibition, allowing for fewer side effects compared to traditional cytotoxic chemotherapy [27]. However, the heterogenous nature of most cancers, the presence of a wild-type gene in addition to the mutated gene, and the capability to develop resistance to targeted therapy has posed problems in the field [28]. As mentioned earlier, melanoma patients treated with PLX4032 developed resistance in about 6–8 months, likely due to acquisition of new mutations in proteins such as N-RAS or MEK, overexpression of Receptor Tyrosine Kinases, or activation of alternative signaling pathways [29,30]. The use of RAF inhibitors such as PLX4032 holds promise because of its initial effectiveness and selectivity, and the hope that combinational therapies or earlier detection methods may eliminate resistance problems in the future. This study supports the application of targeted molecular therapy in thyroid cancer as suggested in earlier studies, by demonstrating that targeting mutated BRAF leads to drastic, selective phenotype changes in thyroid cancer cells in culture [31,32].

Within our mutated BCPAP papillary thyroid cancer cells, inhibition of BRAFV600E impaired survival ability, decreased proliferation, caused apoptosis, and halted DNA synthesis, demonstrating the importance of this signaling molecule in contributing to cancer cell growth. These marked functional effects of BRAF inhibition likely occurred as a result of impaired phosphorylation of downstream molecules within the MAPK pathway including phospho-MEK, phospho-MAPK, and phospho-mTOR. With 10 μM PLX4032 treatment in BCPAP cells we were able to completely abolish expression of phospho-MAPK, which is important because a prior clinical study involving melanoma patients treated with PLX4032 showed that 80% inhibition of phospho-MAPK was required for tumor regression [33]. The dependence of these papillary thyroid cancer cells on signaling through the MAPK pathway and the resulting profound anti-cancer effects by BRAF inhibition can be characterized as “oncogene addiction” [34] and validate targeting the BRAFV600E mutated protein in thyroid cancer cells.

In addition to anti-thyroid effects seen with treatment of the specific RAF inhibitor PLX4032 in BRAF-mutated cancer cells, we observed paradoxical activation of the MAPK pathway in BRAF-wild-type Nthy-ori 3–1 thyroid cells, as previously seen in BRAF-wild-type melanoma cells [35]. This effect may reflect the nature of the drug, in that it is capable of binding to wild-type protomers within CRAF-CRAF or CRAF-BRAF dimers and transactivating the associated protomer, and is important in that it could play a role in drug resistance and toxicity [19-21]. PLX4032-mediated increase in MAPK signaling depends on upstream signals from RAS which explains why it is not seen in BRAFV600E mutated cancer cells, however mutations or overexpression of RAS and other upstream molecules may allow for PLX4032-mediated MAPK activation and for acquired resistance by signaling through wild-type dimers in cancer cells [19-21]. This scenario could occur in thyroid cancer as both our papillary thyroid cancer cells and normal thyroid cells express CRAF protein. It should be noted, however, that this MAPK activation did not lead to a significant enhancement of cell proliferation that would be evident by increased DNA replication or cell cycle status. The presence of paradoxical MAPK activation in thyroid cells further emphasizes that selecting thyroid cancer patients for PLX4032 treatment should be dependent on presence of BRAFV600E mutation.

Overall, the results of this study show that BRAFV600E is a viable therapy target in thyroid tumors harboring this specific mutation and in general highlights the potential in exploiting genetic lesions that lead to overactive signal transduction. The effects of inhibiting kinase activity of a single molecule within the MAPK pathway are potent enough to dramatically and specifically alter papillary thyroid carcinoma cellular phenotype, at least *in vitro*. Within targeted molecular therapy, searching for mutated kinases that are associated with poorer prognostic outcomes will provide targets that can be inhibited with maximal effect. As seen in melanoma patients, resistance to BRAF inhibitors such as PLX4032 could occur in thyroid cancer patients as well. To address this, we recommend further investigation into combinational therapies including BRAFV600E inhibitors. The exciting, recent success of a Phase III clinical trial involving Sorafenib, which caused tumor regression in 12% of patients and significantly improved progression-free survival time in patients with advanced thyroid cancer, supports use of kinase inhibitors. Sorafenib inhibits the tyrosine kinases VEGFR and PDGFR in addition to RAF, although it is more selective for CRAF. Recent studies highlighting the potential of combination therapy suggest a synergy between PLX4032 and Akt inhibitors or HER kinase inhibitors in thyroid cancer [36,37]. Further investigation into combination therapy including a specific inhibitor of BRAFV600E could provide treatment advances for BRAFV600E-positive advanced thyroid cancer patients.

## Conclusions

Targeting the specific mutated kinase, BRAFV600E, within papillary thyroid cancer effectively inhibits growth-promoting characteristics of papillary thyroid cancer cells harboring this mutation *in vitro* by preventing phosphorylation of downstream targets and does not have these effects on normal thyroid cells. Although phenotypic changes in normal BRAF-wild-type thyroid cells did not occur upon exposure to the BRAFV600E inhibitor PLX4032, the drug upregulated phosphorylation of MAPK proteins downstream of BRAF including phospho-MEK, phospho-MAPK, and phospho-mTOR. In order to decrease possible resistance through wild-type BRAF signaling and other pathways, this study supports further investigation of combination targeted therapy including a BRAF600E inhibitor in the treatment of BRAFV600E-positive thyroid cancer patients. Further, we conclude that PLX4032 should be used only in carefully screened patients harboring the BRAFV600E mutation.

## Competing interests

The authors declare that they have no competing interests.

## Authors’ contributions

EKH, SR, RS, NT, AG, RB, RKT conceived and designed the proliferation, immunofluorescence, and Western blot experiments. EKH, SR, NT, and RB performed these experiments. EKH, ZD, HZ, RKT planned and performed the EdU uptake assay. EKH, SR, RS, AG, EJS, JG, RKT analyzed the data. EJS, JG, RKT, ZD contributed materials and reagents. EH, SR, ZD, RKT wrote the manuscript. All authors read and approved the final manuscript.
